# Anthropogenic Anoxic History of the Tuvalu Atoll Recorded as Annual Black Bands in Coral

**DOI:** 10.1038/s41598-020-63578-4

**Published:** 2020-04-30

**Authors:** Nobuko Nakamura, Hajime Kayanne, Yoshio Takahashi, Michinari Sunamura, Go Hosoi, Hiroya Yamano

**Affiliations:** 1 Department of Earth and Planetary Science, The University of Tokyo, Tokyo, Japan; 2 Faculty of Science and Technology, Keio University, Tokyo, Japan; 3 The Ocean Policy Research Institute, THE SASAKAWA PEACE FOUNDATION, Tokyo, Japan; 4 Collaborative Research Institute for Innovative Microbiology, The University of Tokyo, Tokyo, Japan; 5 Dentsu Inc., Tokyo, Japan; 6 Center for Environmental Biology and Ecosystem Studies, National Institute for Environmental Studies, Tsukuba, Japan

**Keywords:** Stable isotope analysis, Environmental impact

## Abstract

Atoll islands are small, low-lying and highly vulnerable to sea level rise (SLR). Because these islands are fully composed of the skeletons from coral reef creatures, the healthy coral ecosystem plays a pivotal role in island resilience against SLR. The environmental deterioration of reefs caused by increases in the human population has been recently reported, but the timing and process are unknown. We investigated the annual black bands in a coral boring core from Fongafale Island, the capital of Tuvalu, which is a symbolic atoll country that is being submerged due to SLR. The iron redox state and microbial gene segments in the coral skeleton might be new environmental indicators that reveal the linkage between anthropogenic activity and coral reef ecosystems. Our findings provide the first demonstration that iron sulfide has formed concentrated black layers since 1991 under the seasonal anoxic conditions inside coral annual bands. Since the 1990s, increasing human activity and domestic waste-induced eutrophication has promoted sludge and/or turf algae proliferation with the subsequent seasonal destruction, resulting in sulfate reduction by anaerobic bacteria. With the recent climate variability, these anthropogenic effects have induced the mass mortality of branching corals, deteriorated the coral reef ecosystem and deprived the resilience of the island against SLR.

## Introduction

Atoll islands form on coral reef platforms, onto which coral gravel and foraminifera sand are thrown up by waves to a low altitude of 1–4 m. Therefore, the ecosystem processes of coral reefs drive the potential of atoll islands to withstand sea level rise (SLR)^1^. However, land-based pollution and human activity caused by increases in the human population has deteriorated the ecosystem in some capital islands of atoll countries, such as the Marshall Islands, Kiribati, Tuvalu and the Maldives^2–5^.

**Figure 1:**
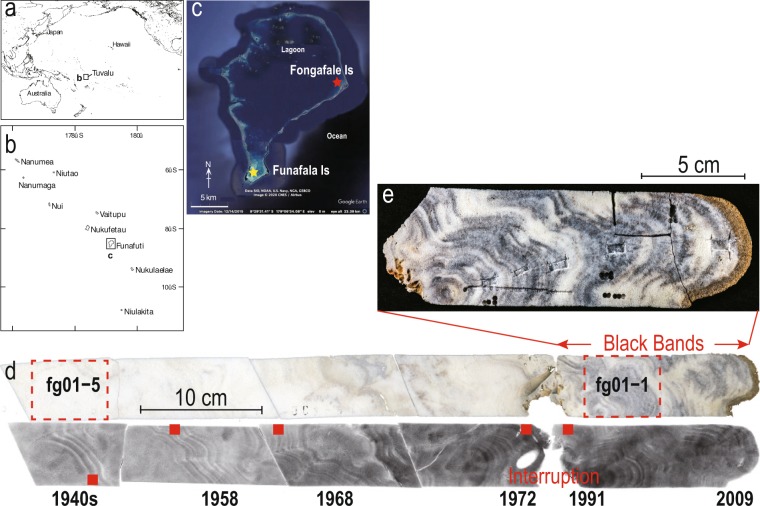
(**a,b**) Maps of Tuvalu and Funafuti Atolls, (**c**) Locations of the two coral sampling sites (Red star; Fongafale Island. fg01, Yellow star; Funafala Island. fh11). The maps were generated from (**a,b**) GSHHG (https://en.wikipedia.org/wiki/GSHHG) using Canvas version 5.0 in Yamano *et al*.^2^, (**c**) Google Earth (Data SIO, NOAA, U.S. Navy, NGA, GEBCO, Image © 2020 CNES/Airbus) using Adobe Illustrator CS5, (**d**) Cross-section of a massive *Porites* coral core from Fongafale Island (fg01); real image (top) and soft X-ray photograph (bottom). The two red dotted frames (fg01–1, fg01–5) represent the sampling areas for the ICP-MS and organic δ^13^C_org_ analyses. The five red squares on the X-ray photograph represent the positions used for the ∆^14^C analysis. (**e**) Black bands, which appear after growth interruption.

**Figure 2:**
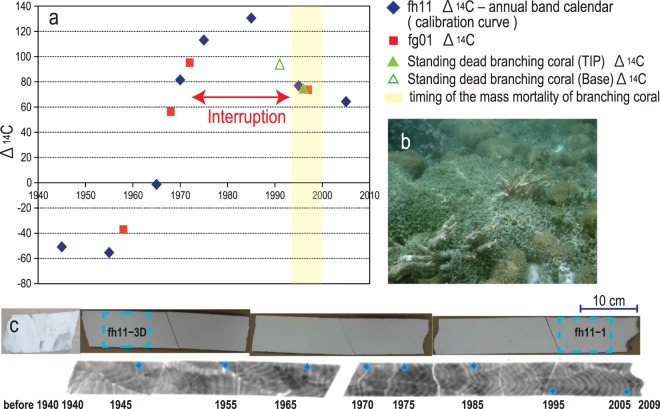
(**a**) Calibration curve for the ∆^14^C values of the coral annual bands derived from fh11 (blue diamonds) and application to fg01 (red squares) and the ∆^14^C value at the tip and the base of the standing dead branching coral (green triangle). The yellow shading indicates the presumed period during which the branch corals experienced mass mortality. (**b**) Standing dead branching corals in the Fongafale lagoon. Photo taken by Prof. Kayanne. (**c**) Cross-section of a massive *Porites* coral core from Funafala Island (fh11): real image (top) and soft X-ray photograph (bottom). The two blue dotted frames on the real image (fh11–1, fh11–3D) represent the sampling areas used for the ICP-MS and organic δ^13^C_org_ analyses. The eight blue squares on the X-ray photograph represent the positions used to generate the ∆^14^C calendar.

Tuvalu is located in the tropical South Pacific and consists of nine atolls (Fig. 1a,b). Fongafale Island, the capital island of Tuvalu, which is an atoll country that has frequently been reported by popular media because it is being submerges due to SLR, is located on the eastern side of the Funafuti Atoll (Fig. 1c). At present, the highest elevation on Fongafale Island is that of the oceanward storm ridge, which has a maximum altitude of ~5 m above the mean sea level (MSL)^2^. The storm ridge is constructed from coral rubble-like branches of *Acropora* coral that have been transported by storms^2^. This small island, which is only 700 m at its widest point, is densely populated with almost 5,000 inhabitants^6^. From the coastal water and sediment analyses in 2010–2011, the high concentrations of *Escherichia coli*, acid volatile sulfide (AVS) and heavy metals due to human sewage and waste were detected^3,4^. This lagoon water pollution in Fongafale Island caused a decrease in the foraminifera population in front of the lagoonal coast of Fongafale Island^3,4^. Lovell *et al*. (2004) mentioned the decline of the coral cover and the immigration of turf algae due to human activity (Destructive fishing) and strong waves in the western side of the atoll^5^. In 2009 almost all branching corals in front of Fongafale are standing dead and covered by green or brown algae and cyanobacteria (Fig. 2b). Macroalgal blooms (*Sargassum polycystum*), which exhibit a positive correlation with high nitrate levels in the lagoon, have been reported since 2011^7,8^ and have resulted in the decay of corals along the coast. However, the lack of historical and long-term observations of this pollution and the actual data of seawater quality before 2009 impedes an understanding of the timing and process of ecosystem deterioration.

Massive corals (e.g., *Porites lutea*) provide excellent paleoclimatic and paleoenvironmental records in their aragonite (CaCO_3_) skeletons^9^. *Porites* corals grow at a rate of 1–2 cm/year, and seasonal variabilities in the sea-surface temperature (SST) have induced the formation of annual density bands in the skeleton^10^. The coral stable oxygen isotopic ratio (δ^18^O_coral_) in carbonate skeletons is used as proxies for the local SST and sea surface salinity (SSS) determined by the balance between precipitation and evaporation^9,11–13^. The fluorescent bands along coral density bands under UV light illumination reflect the organic matter (corrosive acids, including fulvic and humic acids)^14,15^. The intensity and timing of the fluorescent bands on the skeleton are considered proxies for precipitation, river runoff, floods and environmental change caused by human activities^16,17^. The organic stable carbon isotopic ratio (δ^13^C_org_) is generally used as a proxy for nutrient carriers and time-integrated environmental conditions^18,19^. Trace elements, including heavy metals, in coral skeletons have been used as proxies for sediment loading and anthropogenic land use^20,21^. These various proxy measures in coral skeletons record the environmental changes caused by global climate change and local anthropogenic activity.

Here, we investigate and verify the process underlying the effects of human activity on the atoll environment. In March 2009, we obtained coral cores from a massive *Porites* coral and a dead branching coral at two different sites in the Funafuti atoll in Tuvalu (Fig. 1c, Table S1).

## Results and Discussion

### Coral annual bands and chronology

The first coral core was obtained from a rare survivor in the lagoon in Fongafale Island, which was covered by turf algae on the surface (TV09-fg01; 8°31′7.3″S, 179°11′43.9″E, 78 cm). The core has unclear annual bands on an X-ray photograph and experienced a period of interrupted growth (Fig. 1c,d). Another coral core from the Funafala Island lagoon (TV09-fh11; 8°37′48.4″S, 179°04′44″E, 98 cm), which is located 18 km south of Fongafale Island in a relatively unimpacted environment, was collected as a control. The fh11 core exhibits 69-yr continuous annual bands (1940–2009), and these were used to prepare the ∆^14^C bomb calibration curve (Figs. 1c, 2a,c). The chronology was introduced into the interrupted fg01 core as follows: the coral continued to grow from 1991 to 2009 after the interruption, as determined by counting its annual bands from the top to the bottom of the core. The growth of the fg01 coral started in the 1940s and was interrupted once in 1972 ± 0.7, as identified from the coral ∆^14^C analysis (Fig. 2a). Therefore, the annual bands in the fg01 core formed from the 1940s to 1972 ± 0.7 and then again from 1991 to 2009.

**Figure 3:**
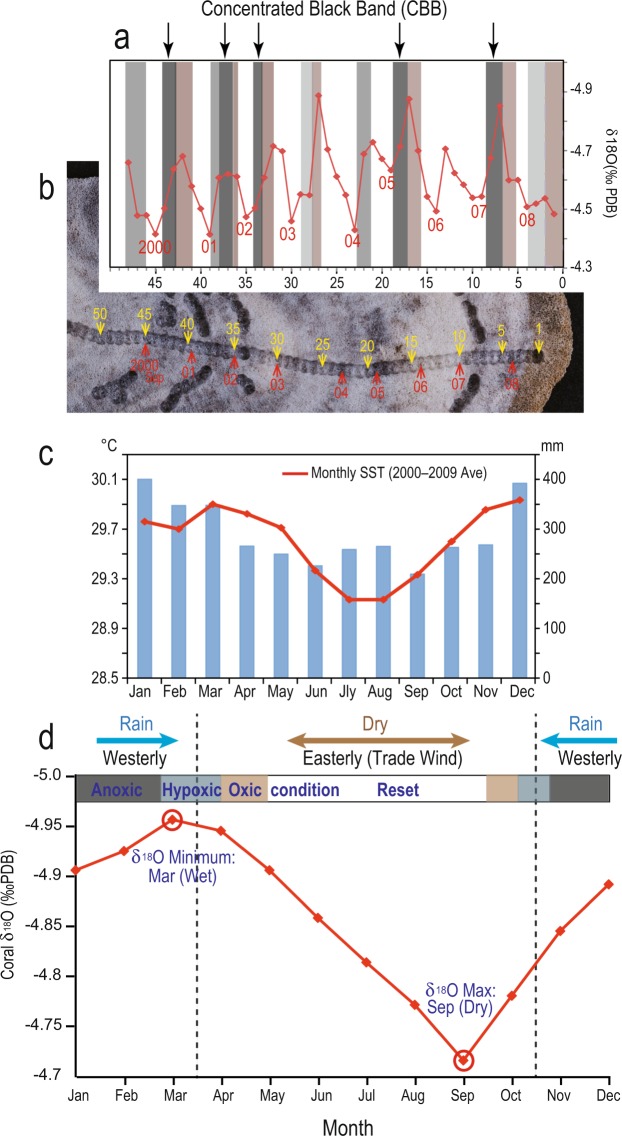
(**a**) Superimposition of the seasonal coral δ^18^O cycle (red line) on the color gradation layers of the fg01 annual bands (2000–2009). (**b**) Trace of the subsamples used for δ^18^O analysis (yellow sequential numbers #1–50). The position of the red number corresponds to the maximal δ^18^O value, which was associated with September. (**c**) Monthly mean SST (2000–2009 monthly mean; red line) and precipitation (1993–2008 monthly mean; blue bar) in Tuvalu. (**d**) Seasonal age model based on the monthly mean coral δ^18^O obtained for 1978–2009 (monthly δ^18^O data for fh11; 1 σ: 0.03‰). The maximal (minimal) δ^18^O value corresponds to September (March), which is the month with the lowest (highest) SST and precipitation in Tuvalu. The color gradation layers in Fig. 2a appear from the Austral spring to early fall seasons in a coral annual band.

**Figure 4:**
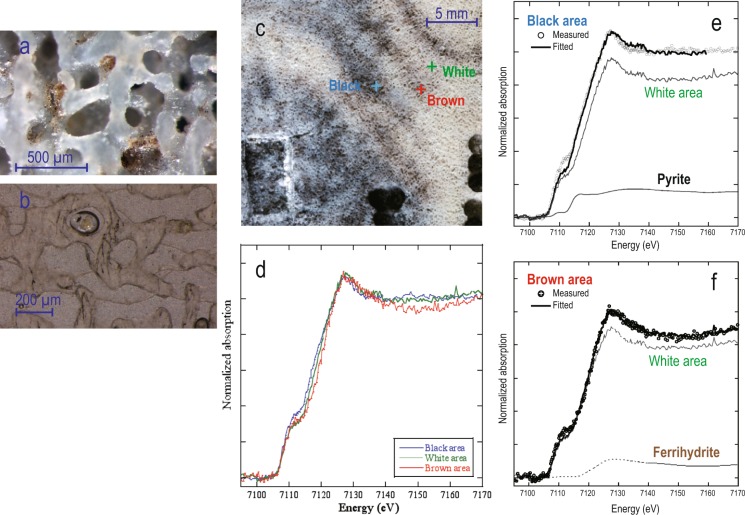
(**a,b**) Photographs of the thin slab section of fg01–1 under an optical microscope. Black and brown microgranules (**a**) and black fibers (**b**) were observed only within the CBBs. (**c**) Locations of the X-ray absorption fine structure (XANES) measurement (three crosses: black, reddish-brown and white area). (**d**) XANES spectra of the black (blue line), reddish-brown (red line) and white (green line) areas. (**e,f**) Each spectrum was fitted based on linear fitting of the spectra of the white area, pyrite (iron sulfide, (**e**) and ferrihydrite (iron hydroxide, **f**). The color gradation of the CBBs indicates that these areas formed due to the redox state of iron.

The coral annual bands that formed after recovery (since 1991) present distinctive black layers (bands) in the white skeleton (Fig. 1e). These black bands exhibit color gradation from original white to bluish/dark-gray and black and reddish-brown layers at each end (Figs. 3b and 4c). In particular, thick concentrated black bands (CBBs) were observed in 1991, 1992–1995, 1997–1998, 2000–2002, 2005 and 2007 (Fig. 3a,b). The seasonal timing of CBBs, as identified by the age model based on the seasonal cycle of monthly coral δ^18^O data, corresponds to Austral spring–fall (November–March) in the rainy season, which is characterized by westerly winds^22,23^ (Fig. 3a,c,d).

### Dead branching coral and mass mortality

Dead branching corals (mainly *Acropora*) were present around the *Porites* coral (fg01). Almost all of branches covered by green or brown algae and cyanobacteria (Fig. 2b). We obtained seven branches from Fongafale lagoon and performed ∆^14^C measurements from the tips and bases of each branch. The ∆^14^C value was fitted to the above-mentioned ∆^14^C bomb calibration curve, and the ∆^14^C value obtained for the tip revealed that the branching coral experienced mass mortality in 1997 ± 3 (Fig. 2a).

### CBB analyses

The aragonite crystal of the coral skeleton was dyed black in the CBBs (Fig. 4a). The heavy metal elements in the CBBs were detected through an ICP-MS analysis (Fe ≫ Zn > Ni > Cu > Cr > Mn > Pb; Table 1). The concentration of iron (Fe) was substantially higher than that of the other elements in the CBBs (fg01–1), and the concentrations of iron, zinc (Zn) and lead (Pb) in the CBBs were three-fold higher than those in the bottom area corresponding to the 1940s (fg01–5) and those in the Funafala core (fh11) (Figs. 1d and 2c, Table 1). Based on the detection of highly concentrated iron, we deduced a relationship between the iron redox state and the color gradation in the black layers. An X-ray absorption near-edge structure (XANES) analysis was performed to verify the iron redox state inside the microscopic area. The XANES spectra of the iron microspots in three different-colored areas (CBBs, reddish-brown and white, Fig. 4c) revealed two types of Fe formations (Figs. 4d–f, S1): iron sulfide (pyrite) was the most strongly reduced compound (Fe^2+^) in the CBBs, and ferrihydrite was iron hydroxide (Fe^3+^) in the reddish-brown area. The detection of iron sulfide microparticles through a scanning electron microscopy (SEM) analysis supported the results from the XANES analysis of the CBBs.

**Table 1:** Trace element analysis by ICP-MS.

Conc. [mg/L]	Cr	Mn	Fe	Ni	Cu	Zn	Cd	Pb
Std: JCp-1*	0.14	0.52	—	4.2	0.68	0.42	0.03	0.18
Fg 01–1 (Black)	1.3	0.9	43.1	4.2	3.4	7.5	0.1	0.6
Fg 01–1 (White)	1.5	1.3	168.1	5.0	1.8	7.6	0.1	0.7
Fg 01–5	3.1	0.5	26.6	3.6	6.2	2.7	0.0	0.2
Fh 11–1	1.7	0.3	21.3	3.3	3.1	3.8	0.0	0.1
Fh11–3D	0.8	0.5	23.7	4.1	1.8	1.8	0.0	0.1

Coral powder was obtained from the two sampling areas, namely, the top and bottom of the fg01 (two red dotted frames in Fig. 1d are denoted fg01–1 and fg01–5) and fh11 (two blue dotted frames in Fig. 2c are denoted fh11–1 and fh11–3D) cores. fg01–1 (black) represents the CBBs, and fg01–1 (white) represents the space between CBBs, as shown in Fig. 4c. The concentrations of the heavy metal elements included in JCp-1, which was used as a standard coral, were based on the values reported by Inoue *et al*. (2004)^32^. *JCp-1: Inoue *et al*., 2004.

In addition, strong fluorescent bands were observed along the CBBs under UV light (Fig. S2). These fluorescent bands indicated the presence of an abundance of organic matter within the CBBs. The organic carbon isotopic ratio (δ^13^C_org_) in the CBBs (−11.1‰) was approximately 5‰ heavier compared with that in the bottom area corresponding to the 1940s (fg01–5; −15.7‰) and that in the Funafala core (fh11–1; −16.4‰, fh11–3D; −16.9‰) (Figs. 1d and 2c). Furthermore, black fibers resembling microorganisms were also observed specifically in the CBBs under an optical microscope (Fig. 4b). DNA analyses were performed at four different depths of the CBBs (Fig. S3a), and DNA of anaerobic bacteria (*Clostridia* and *Desulfatiglans*, which are putative sulfate-reducing Deltaproteobacteria) was detected in the deeper CBB samples (Fig. S3, Tables S3 and S4).

## Discussion

All of the above-mentioned results suggest that coral CBBs seasonally were formed from heavy metals, particularly iron, under a strong reducing environment since 1991. These metals would seem to be taken from seawater and deposited inside aragonite crystals as a black pigment. Anoxic conditions might have dominated during the process of coral CBB calcification, which resulted in the formation of iron sulfide. The detection of microparticles of pyrite inside CBBs and the identification of DNA of sulfate-reducing bacteria in the deep CBB sites suggest the presence of severely anoxic conditions. After the recovery of oxic from anoxic conditions, iron sulfide gradually oxidized to ferrihydrite, and this iron redox state might be responsible for the formation of color gradation layers from black to reddish-brown (Figs. 3b and 4c).

The excess organic matter within the CBBs and the heavy δ^13^C_org_ value indicate the presence of other organic materials except from the coral host tissue and symbiotic algae (from −16 to −15‰)^18,19^. These organic materials might have served as nutrition for bacteria, and the metabolic consumption of light carbon (^12^C) might be a cause of the relatively heavy δ^13^C_org_ value. On the surface of surplus organic materials like turf algae that cover coral calcification tissue, sulfate reduction might occur by anaerobic bacteria due to precipitation of iron sulfide (pyrite) inside the coral skeleton.

The seasonal timing of CBBs recorded in the coral annual bands after the growth interruption, revealed that anoxic conditions occurred during the westerly rainy season from November to March since 1991 (Fig. 3d). This finding suggests that an anoxic environment formed on the surface of the *Porites* coral just during this season. For example, sludge and/or turf algae might be trapped in an indentation of the coral surface during the Austral spring–fall season and can later be removed. In 2011, serious algal proliferation was reported from Sep to Nov; the algae drifted ashore by westerly winds, and its decomposition caused a putrid smell (likely caused by H_2_S) and ooze on the Fongafale coast^7^. After the westerly wind reversed to easterly wind during Mar–Oct, the drifting organic matter was pushed away from the coast, which reset the coastal environment to a healthier state. A similar cover of sludge and/or turf algae might cause seasonal anoxic conditions on the fg01 coral surface. This repetition induced the formation of seasonal CBBs in coral annual bands.

Around the *Porites* coral (fg01), the mass mortality of the branching coral occurred in 1997 ± 3 (Fig. 2). This period matched both the timing of fg01-CBB initiation and the increase in the population in Tuvalu^2^. Since the 1970s, the Funafuti population has increased markedly at two stages: a rapid increase (1972–1991, 15–43% of the total Tuvalu population) and a slower increase (1991–2002, 43–47% of the total population). The second phase of population increase corresponds to the timing of the promotion of seasonal strong reduced condition, and the mass mortality of the branching coral in the Fongafale lagoon. The following linkage is suggested: Increase of human activity and domestic waste bring eutrophication and ecosystem transition from healthy coral reefs to turf algae proliferation in the lagoon^5^. Extreme algae proliferation with subsequent destruction could cause the strong reduced condition and push the anaerobic bacteria activity. The supply of iron to Fongafale Island dates back to WWII, when tanks and equipment for the construction of a runway were delivered^24^. Since the 1990s under the anthropogenic anoxic conditions, iron sulfide-CBBs were formed in coral.

Recent climate change represents an additional threat to the coast. The largest ENSO event (1997–1998) in the 20^th^ century affected precipitation in the Pacific Islands^23,25^. In 1999–2000, Tuvalu recorded a historical precipitation shortage^23^. This heavy drought inhibited water flow in the lagoon, and the resulting stagnation decreased the water quality^7^, as indicated by the particularly thick CBB that formed in the coral skeleton in 2000 (Fig. 3a,b).

### Implication and future investigation

We found the iron redox state and microbial gene segments in the coral skeleton might serve as new environmental indicators, which reveal the linkage between anthropogenic activity and the coral reef ecosystem. This finding could open a new avenue for the study of coral bands because this indicator has not been applied in coral annual band analyses. Comparison with the actual data of seawater quality and further discussion in relation to the biology and physiology of corals are needed to valid and investigate these new proxy measures.

Since 1991, anthropogenic pollution-induced seasonal anoxic conditions have deteriorated the Fongafale coral reef ecosystem. The atoll islands are fully composed of skeletons and shells from coral reef creatures, such as *Acropora* branches, and the healthy reef ecosystem plays a pivotal role in the resilience of an island against climate change^2,26–29^. The ecosystem shift from coral to algal overgrowth due to anthropogenic pollution greatly reduces the high potential of island formation in the atoll islands of Small Island Developing States (SIDS). The restoration of healthy coral reef ecosystems is strongly recommended for increasing island resilience against SLR.

## Methods

### Tuvalu climate

The mean SST is 29.5 °C (with a range of 0.7 °C/yr). The mean precipitation is 3,493 mm/yr, and the climate is characterized by a rainy season with westerly winds from November to March and a dry season with easterly winds from April to October. The SST data was sourced from NOAA Coral Reef Watch^21^ (monthly mean SST for 2000–2009) and the Rainfall data was sourced from Taylor’s Atlas^22^ (the monthly mean precipitation for 1993–2008) (Fig. 3c). Strong El Niño events and/or South Pacific convergence zone (SPCZ) migration disturb the variability in the seasonal precipitation^24^.

### Sampling method and X-radiographs

In March 2009, five coral cores were obtained using an air drill^30^ from three living colonies of *Porites lutea* at two sampling sites, namely, Fongafale Island (TV09 fg01–03; 8°31′7.3″S, 179°11′43.9″E, Fig. 1c,d) and Funafala Island (TV09fh-11 and TV09fh-12; 8°37′48.4″S, 179°04′44″E, Figs. 1c and 2c). The TV09 fg03 core used for the DNA analysis comprises the same living colonies of *Porites lutea* as TV09 fg01 (Table S1). The surfaces of the colonies were found at depths of 1.5 m (fg-01) and 1.7 m (fh-11 and fh-12) in shallow water. The core was cut into a pair of 5-mm-thick slabs, X-radiographs were obtained to determine the annual density bands, and a line along the maximum growth axis was subsampled. A cabinet X-ray system (CMB-2, SOFTEX, Japan) at the Department of Earth and Planetary Science, University of Tokyo, was used to obtain the X-radiographs under the following exposure conditions: X-ray tube voltage of 38 kVp, cathode current of 3.7 mA, and irradiation time of 60 sec.

Chemical instrumental analyses were performed to identify the black contaminant in the fg01 CBBs.

### Fluorescent bands

To identify the organic contamination, a photograph of the fluorescent bands was obtained under shortwave ultraviolet rays (excitation wavelengths of 254 and 365 nm, Fig. S2).

### Coral organic δ^13^C analysis

The coral powder samples (20 mg) from the two sampling areas, namely, the top and bottom of each core [two red dotted frames in fg01 (Fig. 1d) and two blue dotted frames in fh11 (Fig. 2c)] were placed in silver containers and pretreated with hydrochloric acid to remove carbonates as described by Tanaya *et al*. (2018)^18^. Subsequently, 1 N HCl was added until bubbles were no longer observed, and the samples were dried overnight at 60°C and then for 1 h at 105 °C. The dried samples were wrapped in tin foil. We measured the stable carbon isotope ratio using an elemental analyzer-connected isotope ratio mass spectrometer (FLASH EA 1112/DELTA^Plus^ Advantage, Thermo Electron, Inc.) at Port and Airport Research Institute. The δ^13^C value per million is reported as the relative deviation from the VPDB. The analytical precision of the isotope ratio mass spectrometer, which was based on the standard deviation of the δ^13^C values of internal reference replicates, was <0.2‰.

### Coral ∆^14^C measurement (age determination)

We first constructed the ∆^14^C bomb calibration curve for the Funafuti atoll using the continuous coral annual bands (fh11) obtained from Funafala Island south of the Funafuti lagoon. The eight samples of the fh11 core used for the ∆^14^C measurement were obtained from eight positions, and the date of each position was determined by counting the continuous annual bands every 5 years from 2005 to 1945 (Fig. 2a,c). The plotting of the ∆^14^C values per calendar year yielded the ∆^14^C bomb calibration curve. Subsequently, the ∆^14^C values obtained for five samples, including both edges of the growth interruption reflected by the noncontinuous annual bands in fg01, were measured and analyzed using the above-mentioned ∆^14^C bomb calibration curve to obtain the calendar year (Figs. 1d and 2a).

The coupled tips and bases of seven dead branches of *Acropora* (each approximately 10 to 20 cm in length, Fig. 2b) were then measured. Their ∆^14^C values were correlated with the ^14^C bomb curve to obtain the time of death. The ^14^C measurements were conducted at the University of Tokyo, Japan. The ∆^14^C values (‰), along with their decay, were calculated according to the standards defined by Stuiver and Polach (1977)^31^.

### Monthly coral δ^18^O analysis and the seasonal age model

After ultrasonic cleaning, the powdered samples (fg01–1, fh11) used for measuring the δ^18^O values were drilled from the core at 1.5-mm intervals (almost a monthly scale, Fig. 3b) using a microsampling machine along the maximum growth axis. The stable isotope analyses were performed using a Finnigan MAT252 isotope ratio mass spectrometer equipped with an automated carbonate reaction device (Kiel III) at the University of Tokyo. All isotope values were reported with respect to a Pee Dee Belemnite (PDB) standard. A laboratory working standard (JCp-1) was used to translate the raw measurement results into the PDB scale. The external precision of the δ^18^O values of the samples of the powdered carbonate standard was 0.03‰ (*n* = 5, 1 σ).

The seasonal age model was constructed from the monthly mean δ^18^O data obtained for fh11 from 1978–2009. The fh11 core was appropriate for the construction of the age model due to its continuous healthy state. The maximal (minimal) δ^18^O value in a seasonal cycle was anchored as September (March), which was the month with the lowest (highest) SST and precipitation in Tuvalu^21,22^ (Fig. 3c,d). The color of the coral skeleton at each subsampling position used for the δ^18^O measurement was identified by visual inspection (Fig. 3a,b). The information on the color and location for an annual band was superimposed with the seasonal δ^18^O age model (Fig. 3d).

### XRD analysis

The minerals in the coral CBBs were examined by X-ray diffraction analysis. Ten grams of the CBB coral powder was decomposed in 400 mL of acetic acid (1.5 M) over 4 days, and a black residue was obtained. The sample (fg01–1 CH_3_COOH residue) was used for X-ray diffraction analysis [X’Pert PRO MPD (PANalytical) (Cu, 45 kV, 40 mA)] at an angle between 3 and 70° (2θ) at a rate of 2° per minute (Table S2).

### ICP-MS

To determine the concentrations of heavy metals, 100 mg of coral powder from the two sampling areas, namely, the top and bottom of each core [two red dotted frames in fg01 (Fig. 1d) and two blue dotted frames in fh11 (Fig. 2c)], was dissolved in 2 mL of concentrated HNO_3_ (68%) overnight at 180°C. The black residue from fg01–1 was obtained and completely dissolved by the addition of 10 mL of 2% HNO_3_. The solution was evaporated, and the residue was redissolved in 2% HNO_3_. The elements were determined by ICP-MS (Agilent 7700) using indium as the internal standard element. The concentrations of the heavy metal elements included in JCp-1, which was used as a standard coral, were based on those reported by Inoue *et al*. (2004)^32^ (Table 1).

### XANES and μ-XANES

In the analysis of the ICP-MS results, we focused more on the iron content than those of the other elements. We attempted to derive the relationship between the iron redox state and the color gradation in CBBs. A XANES analysis was performed to verify the iron redox state inside the microscopic CBB area.

Iron K-edge XANES analyses were conducted at two beamlines, BL-12C and BL-4A, using an incident X-ray with beam sizes of 0.6 (V) × 0.6 (H) m^2^ and 5 (V) × 6 (H) μm^2^, respectively, at the Photon Factory, KEK (Tsukuba, Japan)^33,34^. A thin section of the coral sample was prepared for the analyses. XANES at BL-12C was employed to estimate the average Fe species within the mm-size band at the surface of the coral sample (Fig. 4d–f). The beam position on the sample was adjusted using an infrared laser beam adjusted to the position of the X-ray beam on the sample to obtain the XANES in normal white areas and in the black and reddish-brown bands. The thin-section sample was fixed to a sample holder oriented at an angle of 45° to the orthogonal direction of the beam. The iron Kα fluorescence X-ray was measured with a 19-element Ge solid-state detector (Canberra, USA).

In contrast, μ-X-ray fluorescence (XRF) mapping and μ-XANES analyses were conducted at BL-4A to obtain the distribution of Fe at a 5-μm spatial resolution (Fig. S1a–e). The incident beam was focused on the above-mentioned size by a Kirkpatrick–Baez mirror system. The sample, which was fixed at an angle of 45° to the orthogonal direction of the beam, was scanned at steps of 5 μm to obtain the μ-XRF mapping data. The fluorescence X-rays of Fe and other elements were measured using a silicon drift detector (Amptek, Inc.). The XRF mapping data were employed to select specific spots in the white, black, and reddish-brown areas (bands) for measurement of the μ-XANES spectra.

### SEM observations

To confirm the existence of pyrite in the CBBs, as indicated in the XAFS results, SEM observations were conducted. The residue after decalcification of the CBBs by acetic acid was prepared for SEM examination. The particles in the residue were coated with carbon tape and analyzed using a Hitachi S-4500 SEM with a cold-field emission gun. The iron sulfide was observed with secondary electron images at an acceleration voltage of 2–10 kV.

### Microbial signatures and DNA analysis

The microbial signatures in the coral skeletons were analyzed based on the SSU rRNA gene sequences. We sampled four parts of the CBBs in the coral skeletons (fg03–1), and the sections were then stored at room temperature (RT) for several years. The four subsamples obtained from the top of the fg03–1 core and from depths of 1.5, 5, and 20 cm were named coral 1, coral 2, coral 3, and coral 4, respectively (Fig. S3a).

After the surfaces of the subsamples were flame-sterilized, the samples were fractured in an aseptic plastic bag with a vice. Environmental DNA was extracted from 0.2 g of the fractured samples using a commercial DNA extraction kit (Power Soil DNA, QIAGEN) according to the manufacturer’s instructions. For the evaluation of contamination during the analytical procedure, we extracted negative-reference DNA from autoclaved glass beads, as described above. The V4 and V5 regions of the SSU rRNA genes in the extracted DNA were amplified by polymerase chain reaction with Gflex DNA polymerase (Takara Bio) and the Univ 530 F and Univ 907 primers^35^, which are Illumina sequencing primers, using the following program: 30 cycles of 96 °C for 25 sec, 52 °C for 45 sec, and 72 °C for 1 min. After adaptor and index sequences were added by further PCR^36^, the purified SSU rRNA gene amplicons were sequenced by MiSeq using 300-bp paired-end reads. The prokaryotic sequences were combined with both P7 and P5 reads, qualified and searched for 97% OTUs using the USEARCH program^37^, and each OTU was classified based on the SILVA123 database^38^ using the QIIME program^39^. For all eukaryotes, only the P7 side sequences were used for the analysis, and after classification, the prokaryotic OTUs were removed. The microbial community structures were stoichiometrically analyzed with the vegan package^40^ in the R program^41^.

The MiSeq dataset was deposited in the DDBJ Sequence Read Archive (DRA) database with the access number DRA007783.

## Supplementary information

Supplementary Information.

Supplementary Table S3, S4.

## Data Availability

All data from the samples and reference materials are available in the supplementary materials.
