# Supplementary material for: Anthropogenic Anoxic History of the Tuvalu Atoll Recorded as Annual Black Bands in Coral
Source: Sci Rep. 2020 Apr 30;10:7338. doi: 10.1038/s41598-020-63578-4 (PMC7193606; doi:10.1038/s41598-020-63578-4)
Supplement: Supplementary file 1 — Supplementary Information. [file 41598_2020_63578_MOESM1_ESM.pdf]

Supplementary Information:

Anthropogenic Anoxic History of the Tuvalu Atoll Recorded as Annual Black Bands in Coral

Nobuko Nakamura[1, 2, 3] *, Hajime Kayanne[1], Yoshio Takahashi[1], Michinari Sunamura[1, 4], Go Hosoi[1, 5] & Hiroya Yamano[6]

[1] Department of Earth and Planetary Science, The University of Tokyo, Tokyo, Japan.

[2] Faculty of Science and Technology, Keio University, Yokohama, Japan.

[3] The Ocean Policy Research Institute, THE SASAKAWA PEACE FOUNDATION, Tokyo. Japan.

[4] Collaborative Research Institute for Innovative Microbiology, The University of Tokyo, Tokyo, Japan.

[5] Dentsu Inc. Tokyo, Japan.

[6] Center for Environmental Biology and Ecosystem Studies, National Institute for Environmental Studies, Tsukuba, Japan.

email: nobunobusango@gmail.com

Supplementary Note: Another Results.

$\Delta^{14}C$ results for the dead branching corals:

The dead branches of corals on the Fongafale lagoon coast had $\Delta^{14}C$ concentrations ranging from 67‰ to 108‰. The continuous $\Delta^{14}C$ bomb curve obtained from fh11 showed increases in the $\Delta^{14}C$ value from the mid-1950s until a peak of 130‰ was reached in 1985, and this peak was followed by a decay period. Careful sampling from the tip and base of the dead branching coral (#7) revealed a higher concentration of $\Delta^{14}C$ in the base than in the tip, which shows that the growth period of the branching coral corresponds to the decay period in the $\Delta^{14}C$ bomb curve (growth period #7 corresponds to 1991±0.8 to 1997±3). Therefore, the similar branching corals around the lagoon experienced mass mortality in 1997 ± 3 (Fig. 2a, b).

XRD results:

The edge of the void along the blackish skeleton was caked with black and brown microgranules (5–10 μm in diameter), which were identified as clay minerals by XRD analysis (Fig. 4a, Table S2). The XRD results from the residue after decalcification of the CBBs by acetic acid showed clay minerals with a low degree of crystallization (7.36° broad), opal-A (22.73° broad) and gypsum (11.65°, 20.82° and 29.01°).

μ-XRF and μ-XANES results:

Although the speciation of Fe in a normal area is unclear, the spectrum of the black-colored band can be explained by the linear combination of the spectra of the normal area and pyrite, which suggests that the black color is derived from pyrite. In contrast, the spectrum of the brown-colored band can be fitted by the spectra of the normal area and ferrihydrite, which reveals that the brown color was formed by the oxidation of pyrite to ferrihydrite.

A micro-XRF analysis was also employed to obtain maps of Ca and Fe (Figs. S1A, b and c), and the findings showed the presence of an Fe-rich area within the coral skeletons, which was likely due to precipitation from water. The micro-XANES analysis for Spots 1 and 2 in Fig. S1 confirmed the presence of pyrite and ferrihydrite within the black and brown bands, respectively. A comparison of the distributions of Fe shown in the μ-XRF map (Fig. S1) between the black and brown areas suggests that Fe precipitation is more widely distributed within the porous structures of coral in the brown band (Fig. S1c), which might be due to the formation of ferrihydrite by the oxidation of pyrite.

Results of the DNA analysis:

The MiSeq read numbers of coral 1, coral 2, coral 3, and coral 4 samples in the classified OTU table were 2,000, 3,223, 6,539 and 14,021, respectively (Table S3). For the coral 1, coral 2, coral 3, and coral 4 samples, the Chao1 OTU richness in 2,000 reads, which was determined based on the minimum number of reads among the samples, was estimated to equal 42.7, 53.2, 52.3, and 42.2, respectively (Table S3). A cluster analysis of the microbial community structures based on the Euclidian distance showed that the coral samples were separated into surface (coral 1 and coral 2), deep (coral 3 and coral 4) and negative reference groups (Fig. S3b). A total of 108 OTUs were determined in our experiment, and 65 of the

108 OTUs were determined from negative references (Tables S3 and S4). To avoid contamination risk from these 65 OTUs, we focused on the OTUs that were not observed in the negative-reference reads. The surfaces of the coral samples contained coral and fungal DNA (Table S4). In contrast, the proportions of Gammaproteobacteria (*Marinobacter*, *Alcanivorax*, and *Halomonas*) were increased in the deeper parts of the samples. Anaerobic bacteria (*Ruminococcaceae* in *Clostridia* and *Desulfatiglans* in putative sulfate-reducing Deltaproteobacteria) were found in the deeper parts of the coral sample (Table S4).

The coral samples used for DNA analysis were preserved for several years at room temperature under dry conditions. Special caution should be taken when discussing the microbial community structures because the preservation of samples induces degradation of the DNA in coral samples and the growth of specific microbes that do not originate from the coral samples. The DNA of fungi at the top (coral 1) might reflect the growth during preservation due to the high organic concentration. However, the coral DNA (*Hexacorallia* in Table S4) detected on the surface of the coral samples showed that the DNA from the original coral samples was conserved in the coral samples during their preservation at RT. In addition, the dry preservation conditions and the poor organic concentrations at the deeper part of the corals (sample corals 3 and 4) are not suitable for the growth of strictly anaerobic microorganisms, such as *Clostridia* and *Desulfatiglans* (Table S4). The DNA of the strictly anaerobic microbes detected in the original deeper coral samples strongly indicated that they grew at the time of precipitation, and the corals were exposed to an anaerobic environment.

Supplementary Table S1: Sample descriptions.

Location	Sampling site	Spices	Sample No.	Analysis
Fongafale Isl.	8°31′7.3"S, 179°11′43.9"E	*Porites lutea*	TV09 fg01	chronology, CBB chemistry
			TV09 fg02	
			TV09 fg03	CBB DNA analysis
Fongafale Isl.	8°31′S, 179°12′E	*Acropora*	#1	
dead branch coral	8°31′S, 179°12′E	*Acropora*	#2	
	8°31′S, 179°12′E	*Acropora*	#2	
	8°31′S, 179°12′E	*Acropora*	#4	
	8°31′S, 179°12′E	*Acropora*	#5	
	8°31′S, 179°12′E	*Acropora*	#6	
	8°31′S, 179°12′E	*Acropora*	#7	mass mortality period
Funafala Isl.	8°37′48.4"S, 179°04′44"E	*Porites lutea*	TV09 fh-11	$\Delta^{14}C$ bomb calibration curve
	8°37′47.1"S, 179°04′44.3"E	*Porites lutea*	TV09 fh-12	

Supplementary Table S2: List of relative X-ray diffraction peaks obtained for fg01-1.

fg01-1 (CH_3COOH residure)		
No.	pos. [°2Th] Cu	
1	7.36	Clay minerals
2	11.65	Gypsum
3	14.85	
4	16.42	
5	20.82	Gypsum
6	22.73	Opal -A
7	26.21	
8	29.01	Gypsum
9	31.23	
10	34.59	
11	40.74	
12	43.48	
13	47.76	
14	51.35	
15	68.78	

Supplementary Table S3: Summary of the analysis of the SSU rRNA genes in the CBBs of fg03-1

The total OTU numbers from all the reads, total coral reads, total negative references, and coral samples without negative references are 108, 87, 65, and 43, respectively.

Samplename	Total reads	2,000-subsampled reads				
	OTU number	reads number	OTU number	Chao1	Ace	Simpson
coral 1	43	2067	42	42.75	43.35447	0.780302
coral 2	53	3223	49	53.2	53.29009	0.8880165
coral 3	51	6539	49	52.33333	51.94041	0.850465
coral 4	50	14021	37	42.25	42.40533	0.7893635
Ncont	47	2017	47	48.5	48.02456	0.911273
reference	61	2431	61	61.75	62.38748	0.8878885

Supplementary Table S4: Ninety-seven-percent OTUs with the taxonomy of the SSU rRNA gene frequency in each sample and a negative reference (Supplementary Dataset Table S4.xls).

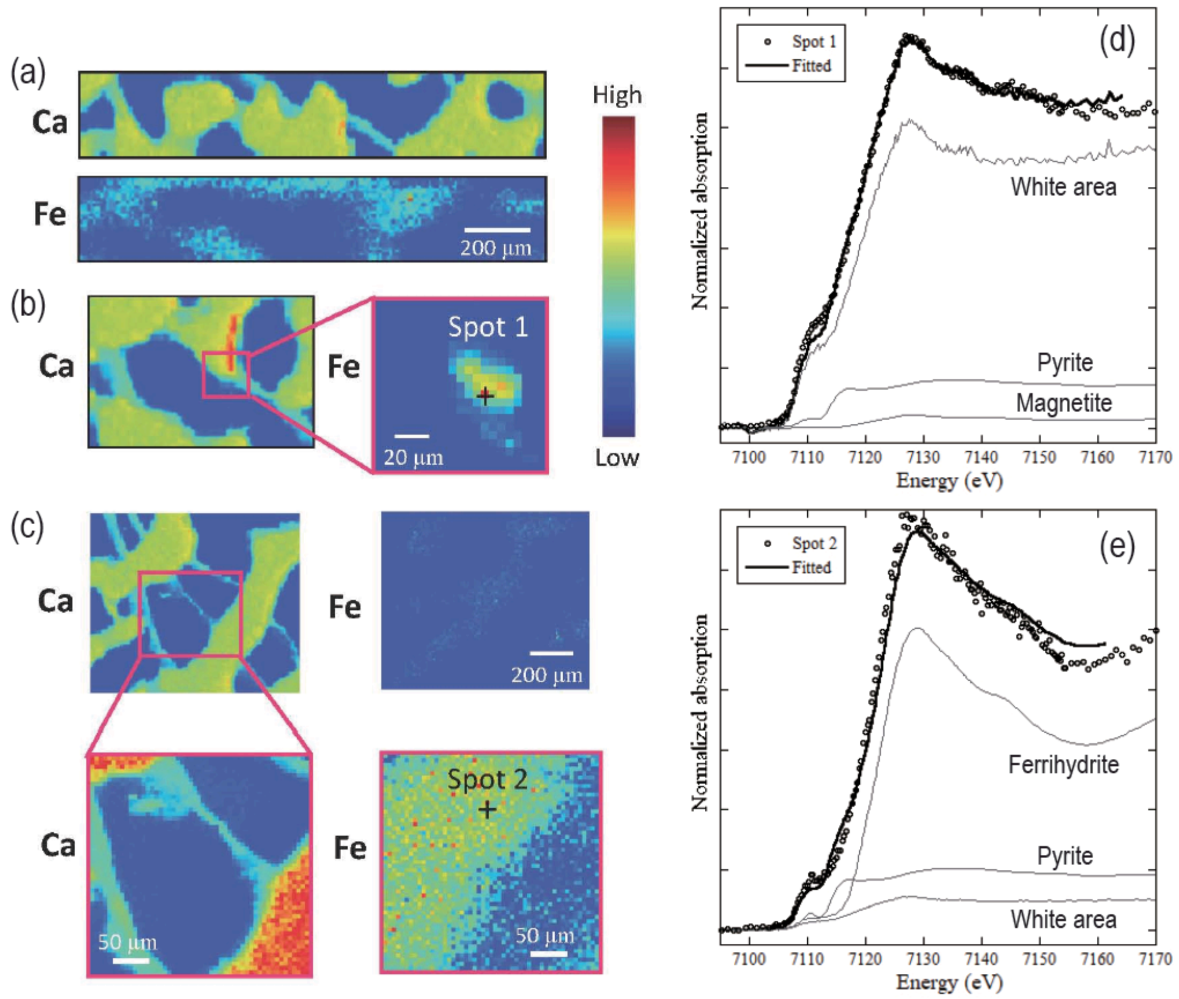

Supplementary Fig. S1: X-ray fluorescence maps of Ca and Fe in the black (a, b) and brown bands (c). The cross shapes labeled “Spot 1” and “Spot 2” denote the positions of the XANES measurements.

A micro-XRF analysis was also employed to obtain maps of Ca and Fe, and the results showed the existence of an Fe-rich area within the coral skeletons, which was likely obtained through precipitation from water. The micro-XANES analysis of Spots 1 and 2 confirmed the presence of pyrite and ferrihydrite within the black and brown bands, respectively. A comparison of the distributions of Fe shown in the μ-XRF map between the black and brown areas suggests that Fe precipitation is more diffuse in the brown bands and the voids of the coral skeleton, which might be due to the formation of ferrihydrite through the oxidation of pyrite.

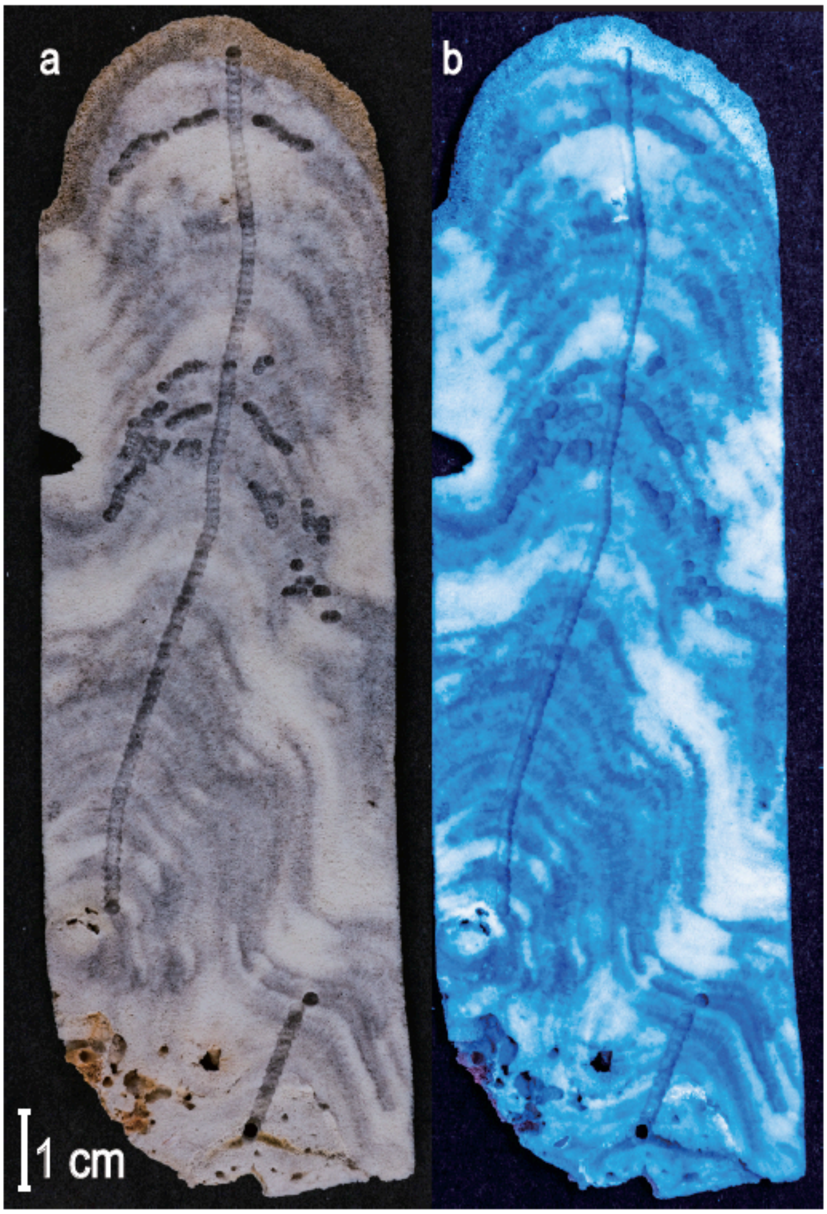

Supplementary Fig. S2: Real image (a) and fluorescent bands of fg01-1 under UV light (b). The images indicate the presence of strong fluorescent bands on the CBB position. Organic matter is hypothesized to originate from sludge, including phosphoric acid, acid volatile sulfide (AVS) [4] and decomposing matter from algal blooms.

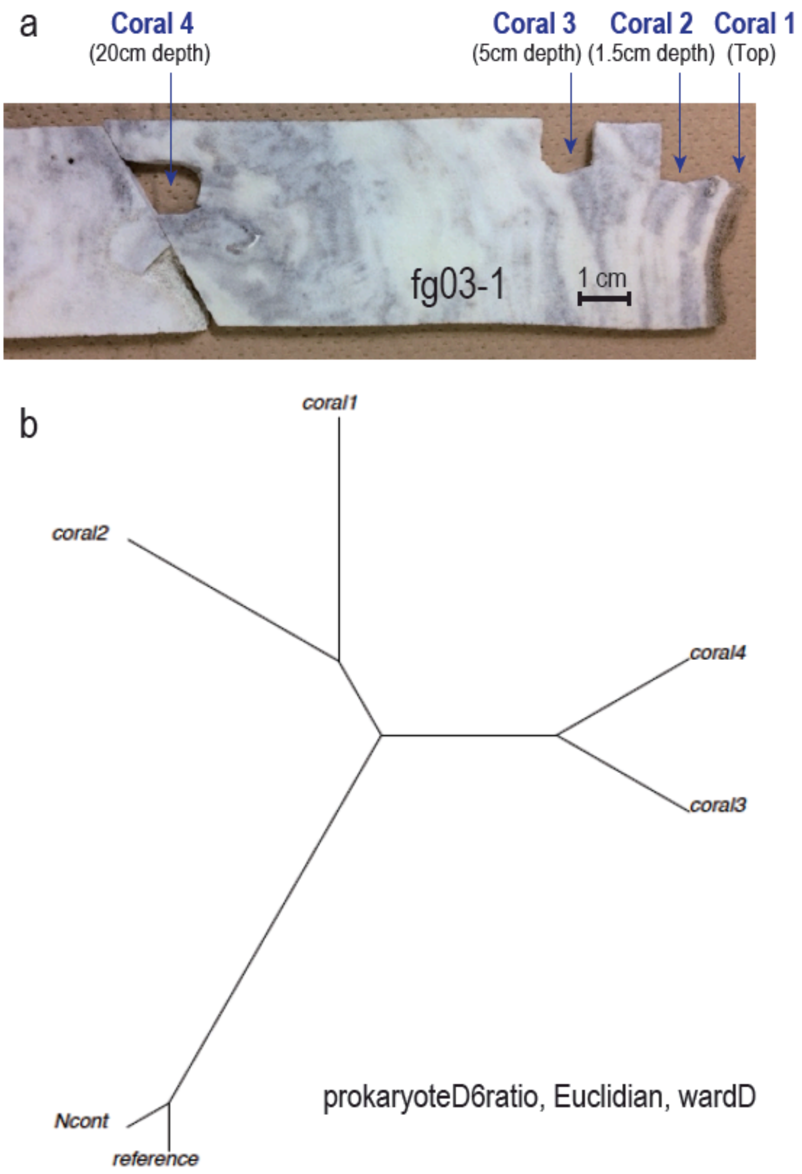

Supplementary Fig. S3: (a) Four subsampling positions in fg03-1 used for DNA analysis: coral 1 (top), coral 2 (depth of 1.5 cm), coral 3 (depth of 5 cm) and coral 4 (depth of 20 cm). The fg03 core was obtained from the same living colonies of *Porites lutea* as TV09 fg01. (b) Clustering dendrogram based on the SSU rRNA gene compositions in the coral skeleton samples (corals 1–4) and negative references (Ncont and reference).
